# Hybrid Lexical–Semantic AI Architecture for Automated Cancer Registry Coding for the Vet-ICD-O-Canine-1 System from Free-Text Veterinary Pathology Reports

**DOI:** 10.3390/cancers18172728

**Published:** 2026-08-23

**Authors:** Vitória Souza de Oliveira Nascimento, Marcello Vannucci Tedardi, Guilherme da Silva Rogério, Katia Cristina Pinello, Maria Lúcia Zaidan Dagli

**Affiliations:** 1 Laboratory of Experimental and Comparative Oncology, Department of Pathology, School of Veterinary Medicine and Animal Science, University of São Paulo, São Paulo 05508-900, Brazil; mvtedardi@gmail.com (M.V.T.); guilherme.rogerio@usp.br (G.d.S.R.); 2 EPIUnit ITR, Institute of Public Health of the University Porto, University of Porto (ISPUP), 4050-600 Porto, Portugal

**Keywords:** artificial intelligence, veterinary oncology, cancer registry, natural language processing, large language models, Vet-ICD-O-Canine-1, ontology-based coding

## Abstract

**Background/Objectives:** Free-text veterinary pathology diagnoses contain essential information for cancer registration but are difficult to convert into standardized ontology-based codes because of linguistic variability, contextual modifiers, and large ontology search spaces. This study evaluated a hybrid lexical–semantic architecture for the automated assignment of Vet-ICD-O-Canine-1 morphology codes. **Methods:** A retrospective single-registry benchmark included 211 diagnoses from the São Paulo Animal Cancer Registry. Of these, 190 contained sufficient morphological information for expert-reviewed reference coding, whereas 21 generic or insufficiently specified descriptions were retained as an exploratory challenge subset. Fuzzy lexical matching retrieved Top-10, Top-20, or Top-30 candidates from the complete 971-entry morphology ontology, followed by semantic selection using Claude Haiku 4.5 and structured JSON output. Performance and computational efficiency were compared to direct full-ontology inference. **Results:** Among the evaluated fuzzy metrics, *token_set_ratio* achieved the highest Top-30 reference-code retrieval rate of 89.5%. End-to-end exact-match agreement increased from 73.7% with Top-10 to 79.5% with Top-20 and 85.8% with Top-30 (95% CI, 80.1–90.0%). Top-30 generated non-null codes for 93.2% of the 190 evaluable diagnoses and achieved a conditional exact-match agreement of 92.1%. By contrast, the direct full-ontology baseline achieved 71.2% conditional exact-match agreement (42/59) among non-null predictions and 22.1% end-to-end exact-match agreement (42/190) when incorrect predictions, null outputs, and technical failures were considered non-concordant outcomes. Compared to direct full-ontology inference, Top-30 reduced input-token consumption by 92.4%, total token consumption by 92.2%, and inference cost by 91.3%, while avoiding the 118 API rate-limit failures observed with the direct baseline. Among the 21 insufficiently specified diagnoses, Top-30 returned null codes in 38.1% and non-null codes in 61.9%. **Conclusions:** Ontology-guided candidate reduction improved coding agreement, computational efficiency, and operational robustness within this retrospective single-registry benchmark. However, the reported performance estimates require confirmation in larger independent datasets, and an upstream data-sufficiency or abstention mechanism is needed before prospective operational deployment.

## Simple Summary

Veterinary pathology reports are usually written as free text, which makes it difficult to organize cancer diagnoses into standardized registry codes. This study evaluated a hybrid artificial intelligence approach designed to automatically assign standardized tumour morphology codes from veterinary pathology diagnoses. The method first reduced the number of possible codes using lexical similarity and then used a language model to select the most appropriate code from this smaller set. The approach achieved high agreement with expert-reviewed reference codes while substantially reducing computational requirements compared to providing the complete coding system directly to the language model. However, the system was less reliable when diagnostic descriptions lacked enough information for a specific classification. These findings support the use of artificial intelligence as a decision-support tool for veterinary cancer registries, while highlighting the continued need for expert review and validation in larger, independent datasets.

## 1. Introduction

The increasing availability of free-text pathology reports has created new opportunities for artificial intelligence (AI) to support cancer research, cancer registration, and data-driven oncology through the automated extraction and standardization of clinically relevant information [1,2,3,4]. However, transforming heterogeneous pathology narratives into standardized ontology-based codes remains challenging because of linguistic variability, contextual modifiers, inconsistent terminology, and the large search spaces associated with controlled vocabularies [1,2,4]. These limitations directly affect data quality, interoperability, and the secondary use of pathology data, restricting the scalability of cancer registries and downstream computational applications [1,2,3,4].

These challenges are particularly relevant in veterinary oncology. Naturally occurring neoplasms are a major cause of morbidity and mortality in companion animals and represent an increasingly important area of veterinary medicine [5]. Beyond their direct impact on animal health and welfare, canine and feline cancers provide valuable models for comparative oncology because they develop under naturally occurring conditions and share clinically relevant characteristics with human malignancies, including similarities in tumour biology, progression, metastasis, and therapeutic response [6,7,8,9]. Companion animals may also act as sentinels of environmental exposures affecting both animal and human health [10]. Consequently, the availability of standardized, interoperable, and reusable veterinary cancer data is essential for cancer registration, epidemiological surveillance, comparative oncology, and translational research [11].

Recent developments in comparative oncology have further emphasized the value of well-characterized canine cancer datasets for cross-species research. Cahill et al. reviewed the expanding genomic resources available for human and canine cancers and highlighted the increasing importance of dogs as naturally occurring models for investigating cancer biology and therapeutic targets [12]. At the same time, Fonti and Millanta identified persistent heterogeneity in companion-animal cancer registration practices and emphasized the need for standardized methods to improve the validity, completeness, and comparability of veterinary cancer data [13].

The systematic classification of veterinary neoplasms is fundamental for comparing tumour characteristics across patients, institutions, populations, geographic regions, and species [11,14]. The Vet-ICD-O-Canine-1 system was developed as a veterinary adaptation of the International Classification of Diseases for Oncology framework to support the standardized coding of canine neoplasms [14]. Nevertheless, veterinary pathology diagnoses are commonly recorded as free-text narratives and vary substantially in terminology, diagnostic detail, language, tumour grade, anatomical descriptors, behaviour, and metastatic information [15,16]. Assigning standardized morphology codes from these heterogeneous descriptions remains a labour-intensive process that depends heavily on expert interpretation and cannot be reliably addressed through exact string matching alone [11,14,15,16].

Large-scale veterinary pathology studies illustrate both the value of these data and the challenges involved in transforming them into structured information. Rodríguez et al. applied text-mining methods to 180,232 free-text electronic pathology records from the United Kingdom, identifying and normalizing 109,895 canine and feline neoplasms to create a pathology-based tumour registry [17]. Similarly, Aupperle–Lellbach et al. retrospectively analyzed 109,616 canine histopathological datasets in Germany, demonstrating the epidemiological value of systematically organized pathology diagnoses for characterizing tumour frequency and breed distributions [18].

The automated extraction and coding of cancer information from pathology narratives have previously been investigated using conventional natural language processing and rule-based approaches. Hammami et al. developed a domain-specific system for assigning ICD-O morphology codes from Italian pathology reports, demonstrating that highly accurate automated coding can be achieved using manually designed linguistic rules [19]. However, rule-based systems may require extensive language-specific development, institutional customization, and maintenance when terminology or reporting practices change. In veterinary medicine, Davies et al. described the progression of clinical text mining from keyword- and rule-based approaches toward machine-learning and language-model methods capable of extracting information from large collections of unstructured veterinary records [20].

Beyond rule-based approaches, machine-learning and deep-learning methods have increasingly been investigated for automated cancer registry abstraction and ICD-O coding. Alawad et al. applied multi-task convolutional neural networks to extract multiple cancer-reporting variables from free-text pathology reports [21]. Rios et al. incorporated the hierarchical structure of ICD-O-3 into a neural multi-task framework for automated topography and histology coding from pathology reports [22]. Mitchell et al. developed the CancerBERT network to extract tumour site and histology information from free-text oncology pathology reports and support their mapping to ICD-O-3 codes [23]. More recently, Villena et al. developed a two-stage approach for Spanish pathology reports in which tumour morphology and topography information was first identified from free text and subsequently mapped to standardized ICD-O codes [24].

More recently, large language models (LLMs) have expanded the range of tasks that can be addressed through the semantic interpretation of free-text clinical narratives [25]. Applications have included diagnostic prediction from veterinary clinical notes [26], automated cancer registry coding from hospital records [27], and ICD-O coding from real-world human pathology reports [28]. Bartels and Carus further evaluated open-source LLMs for structured information extraction from German pathology reports and showed that retrieval-augmented prompting could improve performance, particularly for smaller models [29]. Although these studies demonstrate the potential of LLM-based coding, they also highlight continuing challenges related to computational requirements, generalizability, structured output reliability, and safe integration into operational oncology systems [25,27,28].

A further challenge arises when complete controlled vocabularies or extensive clinical contexts are supplied directly to an LLM. Long-context inference increases token consumption, computational cost, and operational complexity, while relevant information may receive insufficient attention when embedded among numerous competing entries [30]. Retrieval-assisted architectures offer an alternative by separating candidate identification from subsequent semantic reasoning. Retrieval-augmented generation was originally developed to provide language models with a restricted set of relevant external information before generation [31]. Subsequent work has shown that retrieval-based and long-context approaches present different trade-offs and may be combined to improve efficiency and performance [32]. Hybrid retrieval strategies can further integrate complementary lexical, semantic, or structural signals to produce a more relevant inference context [33].

In ontology-based clinical coding, retrieval can serve a related but distinct purpose. Rather than retrieving additional patient information to expand the prompt, a candidate-retrieval stage can reduce the ontology search space by identifying a limited set of plausible concepts before semantic selection. A similar retrieval-assisted principle has recently been evaluated in medical coding, where Anjos de Almeida et al. used semantic retrieval to reduce a large clinical terminology to a smaller set of candidate concepts before asking LLMs to select the most appropriate code [34]. This distinction is particularly relevant for Vet-ICD-O-Canine-1, whose morphology ontology contains hundreds of closely related entries that may compete during unrestricted inference. A hybrid architecture that combines computationally inexpensive lexical retrieval with contextual semantic reasoning may therefore preserve the flexibility of LLM-based interpretation while reducing computational demand and improving operational robustness.

Accordingly, this study aimed to design and evaluate a hybrid lexical–semantic AI architecture for the automated assignment of Vet-ICD-O-Canine-1 morphology codes from free-text veterinary pathology diagnoses. The architecture combines fuzzy lexical candidate retrieval over the complete morphology ontology with LLM-based semantic selection from restricted candidate sets and structured output generation.

The study addressed the following research questions:

(RQ1) Can lexical candidate retrieval effectively reduce the Vet-ICD-O-Canine-1 morphology search space while retaining the expert-reviewed reference code?
(RQ2) Does retrieval-assisted semantic selection improve coding agreement and operational robustness compared to direct full-ontology LLM inference?
(RQ3) How does candidate-list depth affect coding performance, computational requirements, and model behaviour?

The main contributions of this study are as follows:

• A systematic comparison of fuzzy similarity metrics as both standalone classifiers and upstream candidate-retrieval methods;
• An evaluation of Top-10, Top-20, and Top-30 retrieval-assisted semantic-selection configurations against direct full-ontology inference;
• A joint assessment of coding agreement, output availability, model-reported confidence, computational consumption, technical failures, and behaviour on insufficiently specified diagnostic inputs.

The remainder of this article is organized as follows. Section 2 describes the dataset, lexical retrieval strategy, LLM-based semantic-selection architecture, experimental configurations, and evaluation procedures. Section 3 presents the predictive and computational results. Section 4 discusses the findings in relation to previous studies, practical implications, limitations, and future directions, and Section 5 summarizes the main conclusions.

## 2. Methods

### 2.1. Study Design and Dataset

This retrospective benchmarking study designed and evaluated a hybrid lexical–semantic artificial intelligence (AI) architecture for the automated assignment of Vet-ICD-O-Canine-1 morphology codes from free-text veterinary pathology diagnoses. The proposed architecture combines computationally efficient lexical candidate retrieval with large language model (LLM)-based semantic reasoning to generate standardized ontology-based outputs suitable for veterinary cancer registry applications.

The dataset comprised 211 veterinary pathology diagnoses collected by the São Paulo Animal Cancer Registry [35], with manually assigned and expert-reviewed Vet-ICD-O-Canine-1 morphology codes serving as the reference standard. The diagnostic records originated from multiple veterinary pathology laboratories and were issued by multiple pathologists, thereby incorporating inter-laboratory and inter-pathologist variation in diagnostic terminology. The exact number of contributing laboratories and pathologists was not available for the present analysis.

The records were obtained from the São Paulo Animal Cancer Registry and originated from the city of São Paulo, São Paulo State, Brazil. The exact beginning and ending dates of record collection were not available in the dataset provided for the present analysis, which contained only the diagnostic information required for morphology coding.

The original diagnostic text was preserved without manual normalization, token cleaning, or removal of qualifiers, tumour grades, anatomical descriptors, metastatic indicators, or other contextual information. This design enabled the evaluation of the proposed architecture under conditions representative of routine veterinary pathology reporting and cancer registry workflows.

Twenty-one records containing generic or non-specific diagnostic descriptions that did not permit unambiguous morphology coding were excluded from reference-standard agreement analyses but were retained for output-availability analyses. Consequently, 190 records were included in reference-standard agreement and candidate-retrieval analyses.

No a priori sample-size calculation was performed because the study was designed as a retrospective benchmarking evaluation rather than a hypothesis-testing clinical study. The evaluable diagnoses were used to compare predefined lexical similarity metrics and candidate-list depths and to select the final architectural configuration. Neither component of the architecture was trained on these records: the RapidFuzz lexical similarity metrics are deterministic and do not involve supervised learning or parameter fitting, and the pretrained Claude Haiku 4.5 model was used without fine-tuning on the study dataset.

Among these 190 evaluable records, the diagnostic texts comprised 170 unique descriptions distributed across 74 distinct morphology codes, reflecting the lexical diversity and terminological variability encountered in routine veterinary pathology reports. These 74 morphology codes represented the coverage of the reference-standard benchmark and did not define the set of codes accessible to the architecture. During lexical retrieval, each diagnosis was compared against the complete 971-record multilingual computational representation derived from the Vet-ICD-O-canine-1 morphology terminology; however, performance could only be empirically evaluated for morphology codes represented by expert-reviewed reference cases in the benchmark dataset. Only the free-text diagnostic description was processed by the architecture; no identifiable information related to animals or owners was accessed or included during any stage of the analysis.

### 2.2. Fuzzy Matching and Candidate Retrieval

The Veterinary International Classification of Diseases for Oncology Canine Tumours First Edition (Vet-ICD-O-canine-1) was used as the source coding system [14]. The morphology terminology was obtained from the supplementary material accompanying the original Vet-ICD-O-canine-1 publication (accessed on 13 May 2026) [14]. For the computational implementation, the original terminology was retained and Portuguese and Spanish translations were added while preserving the corresponding Vet-ICD-O-canine-1 morphology codes and coding structure. The resulting multilingual CSV file was subsequently converted into the JSON representation used in the experiments, comprising 971 records. Each free-text diagnosis was compared against this computational representation using the RapidFuzz library and four lexical similarity metrics: token_set_ratio, token_sort_ratio, partial_ratio, and WRatio.

For standalone fuzzy classification, the highest-ranked candidate (Top-1) was considered the final prediction. For candidate retrieval, performance was evaluated at Top-1, Top-3, Top-5, Top-10, Top-20, Top-30, Top-50, and Top-100. Retrieval was considered successful when the manually assigned Vet-ICD-O-Canine-1 morphology code was present within the corresponding Top-*N* candidate set. This range was evaluated to characterize the effect of candidate-list depth on reference-code recovery independently of the subsequent LLM-based semantic-selection stage.

Based on the retrieval results, token_set_ratio was selected as the lexical retrieval component of the proposed hybrid architecture. Although token_sort_ratio achieved the highest Top-1 classification accuracy, token_set_ratio demonstrated superior retrieval performance from Top-10 onward, making it more suitable for its intended role of reducing the ontology search space while preserving semantically relevant candidate codes for subsequent LLM-based semantic selection. This strategy enables the LLM to operate over a substantially smaller and more relevant candidate set, improving computational efficiency without restricting semantic interpretation.

### 2.3. LLM Classification and Experimental Configurations

Semantic candidate selection was performed using Claude Haiku 4.5 (model identifier: claude-haiku-4-5-20251001; Anthropic), accessed through the Anthropic API, with a temperature of zero and a maximum output length of 300 tokens. Claude Haiku was selected as the semantic-selection model for this benchmarking study because it supported reproducible API-based inference with structured outputs while allowing repeated evaluation of the proposed architecture at a manageable inference cost. The objective was to evaluate the effect of ontology-guided candidate reduction rather than benchmarking different LLMs.

The system instruction specified that the model would receive a free-text veterinary diagnosis in Portuguese, English, or Spanish together with a list of candidate Vet-ICD-O-canine-1 records and should return only a valid JSON object without additional explanatory text. The user-message template consisted of the original diagnostic text followed by the candidate list serialized in JSON format. The required output fields were codigo (morphology code), nome_padronizado (standardized name), sistema (system), and confianca (model-reported confidence: high, medium, or low). When no candidate was considered suitable, the model was instructed to return null values for the code, standardized name, and system fields, with low confidence. The same prompt structure and output schema were maintained across all LLM-based configurations.

All LLM-based experiments used the same model version, prompt structure, system instructions, and output constraints to ensure that differences in performance were attributable exclusively to the candidate-retrieval stage. This controlled comparison strategy is consistent with recent LLM benchmarking studies that standardized prompt formats or maintained fixed retrieval and prompting conditions across evaluated models or experimental configurations [29,34]. The model was instructed to return a structured JSON object containing a Vet-ICD-O-Canine-1 morphology code and a categorical confidence label (high, medium, or low), or a null code when no suitable candidate was identified.

**Figure 1:**
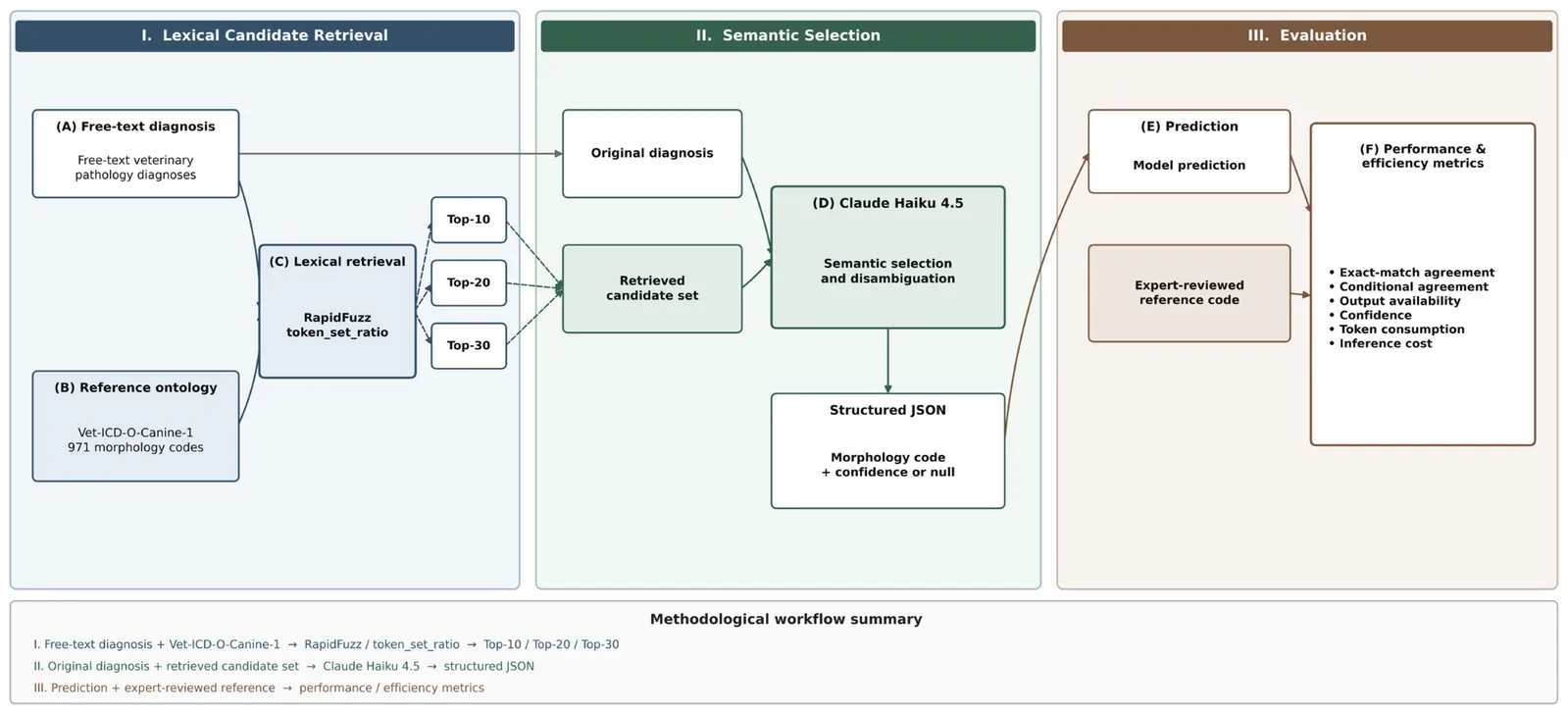
Hybrid lexical–semantic architecture for automated Vet-ICD-O-Canine-1 morphology coding from free-text veterinary pathology diagnoses.

To evaluate the contribution of each architectural component, four experimental configurations were implemented. In the direct baseline configuration, each diagnosis was submitted together with the complete Vet-ICD-O-Canine-1 ontology comprising 971 morphology codes. In the hybrid configurations, the token_set_ratio retrieval stage identified the Top-10, Top-20, or Top-30 most lexically similar candidate codes, which were subsequently provided to the LLM along with the original diagnosis for semantic selection. These progressively larger candidate sets were selected to evaluate the trade-off between candidate coverage and inference-context size while retaining a substantial reduction relative to the complete ontology. Top-30 emerged as the best-performing configuration among the three hybrid candidate-list depths evaluated in this benchmark. The overall hybrid lexical–semantic architecture is illustrated in Figure 1.

All 211 pathology diagnoses were processed under each experimental configuration. No manual correction, filtering, or post-processing of model outputs was performed prior to evaluation.

The proposed workflow combines lexical candidate retrieval with LLM-based semantic code selection. Free-text veterinary pathology diagnoses were processed using the Vet-ICD-O-Canine-1 morphology ontology. Of the 211 original records, 190 had an evaluable expert-reviewed reference code and were included in the primary agreement analyses. The lexical retrieval stage applied RapidFuzz token_set_ratio to the 971-entry morphology ontology to identify the Top-10, Top-20, or Top-30 most similar candidate codes. These candidate sets, together with the original diagnosis, were then provided to the LLM for semantic reasoning and morphology code selection. The structured output consisted of a Vet-ICD-O-Canine-1 morphology code accompanied by a model-reported confidence label or a null response. Model predictions were compared to the expert-reviewed reference standard to evaluate end-to-end exact-match agreement, conditional exact-match agreement, output availability, model-reported confidence, token consumption, and inference cost.

### 2.4. Performance and Efficiency Evaluation

Performance evaluation considered both predictive accuracy and computational efficiency to assess the suitability of the proposed architecture for automated cancer registry coding.

Candidate-retrieval performance was calculated as the proportion of the 190 evaluable records for which the expert-reviewed reference morphology code was present within each Top-*N* candidate set generated during the lexical retrieval stage.

Output availability was calculated across all 211 submitted records as the proportion of non-null model outputs. Null outputs and API failures were recorded separately. API failures, including rate-limit errors, were treated as technical execution failures rather than model abstentions.

End-to-end exact-match agreement was calculated among the 190 evaluable records as the proportion of diagnoses for which the architecture returned a non-null morphology code identical to the expert-reviewed reference standard. Incorrect predictions and null outputs were considered non-concordant outcomes.

Conditional exact-match agreement was calculated only among evaluable records for which the architecture produced a non-null output. Model-reported confidence labels were summarized descriptively and were not interpreted as calibrated probabilities of prediction correctness. To examine the empirical relationship between model-reported confidence and coding agreement, exact-match accuracy was calculated separately for high-, medium-, and low-confidence outputs within each hybrid configuration. Confidence labels were evaluated descriptively and were not subjected to probabilistic calibration.

To provide a broader multiclass evaluation, macro-averaged and support-weighted precision, recall, and F1-score were calculated across the 74 reference morphology codes represented in the benchmark. Null outputs were treated as non-concordant outcomes but were not considered morphology classes for precision, recall, or F1-score calculations. Cohen’s kappa was calculated across all 190 evaluable records, with null outputs retained as a distinct predicted outcome.

For the final Top-30 configuration, component-level error attribution was additionally performed for the non-concordant outcomes. Each non-concordant prediction was classified according to whether the expert-reviewed reference morphology code was present or absent from the retrieved Top-30 candidate set. Outcomes occurring despite successful retrieval were further classified as incorrect non-null predictions or null outputs.

For each LLM-based configuration, input tokens, output tokens, total token consumption, and inference cost were recorded from the model usage metadata. Relative reductions in total token consumption and inference cost achieved by the final hybrid configuration were calculated with respect to the direct full-ontology baseline.

For the principal proportion-based performance estimates, 95% confidence intervals were calculated using the Wilson score method.

### 2.5. Software, Reproducibility, and Ethics

The hybrid lexical–semantic architecture was implemented in Python 3.14.0 using pandas 2.3.3 for data manipulation, RapidFuzz 3.14.3 for lexical similarity calculations, and httpx 0.28.1 for direct HTTP requests to the Anthropic API. The analyses were conducted on a computer running Windows 11 Home Single Language, equipped with an 11th-generation Intel^®^ Core™ i5-1135G7 processor and 8 GB of RAM. No GPU acceleration was used.

To facilitate reproducibility, all experimental configurations were executed using the same software implementation, model version, prompt structure, system instructions, and inference parameters, differing only in the candidate-retrieval strategy.

To support methodological transparency, the manuscript reports the software environment, model identifier, inference parameters, lexical retrieval procedure, ontology source and pre-processing workflow, output structure, and evaluation criteria used in the experiments. The complete source code and associated implementation files are not publicly released at this stage because they form part of an ongoing doctoral research project. However, these materials may be made available upon reasonable request to the corresponding author for academic and reproducibility purposes, subject to applicable institutional policies.

Only free-text histopathological diagnoses were processed by the proposed architecture. No information capable of identifying animals or owners was accessed, processed, or disclosed at any stage of the study.

## 3. Results

The results are presented according to the sequential components of the experimental workflow, beginning with lexical candidate retrieval, followed by the evaluation of the retrieval-assisted configurations, computational efficiency and direct full-ontology inference, and finally the exploratory analysis of insufficiently specified diagnoses and selection of the final architecture.

### 3.1. Selection of the Fuzzy-Matching Strategy

A total of 190 veterinary pathology records with expert-reviewed Vet-ICD-O-Canine-1 morphology reference codes were included in the candidate-retrieval analysis. Original diagnostic texts were processed without manual normalization or removal of diagnostic qualifiers, tumour grades, anatomical descriptors, metastatic indicators, or other contextual information.

Four lexical similarity metrics were evaluated: token_set_ratio, token_sort_ratio, partial_ratio, and WRatio. When fuzzy matching was used as a standalone classifier by selecting only the highest-ranked candidate, token_sort_ratio achieved the highest Top-1 exact-match performance, correctly identifying the reference code in 91 of 190 cases (47.9%). token_set_ratio ranked second, with 84 correct Top-1 predictions (44.2%), followed by partial_ratio with 73 correct predictions (38.4%) and WRatio with 64 correct predictions (33.7%).

The relative performance of the similarity metrics changed when fuzzy matching was evaluated as a candidate-retrieval stage within the hybrid architecture. At Top-3, token_sort_ratio retrieved the reference code in 115 cases (60.5%), compared to 110 cases for token_set_ratio (57.9%), 95 for partial_ratio (50.0%), and 86 for WRatio (45.3%). At Top-5, token_sort_ratio again ranked first, retrieving the reference code in 126 cases (66.3%), closely followed by token_set_ratio with 124 cases (65.3%), whereas partial_ratio and WRatio achieved retrieval rates of 53.2% and 47.4%, respectively.

From Top-10 onward, however, token_set_ratio consistently outperformed the other similarity metrics. At Top-10, it recovered the reference code in 144 of 190 cases (75.8%), compared to 139 cases for token_sort_ratio (73.2%), 118 for partial_ratio (62.1%), and 99 for WRatio (52.1%). At Top-20, token_set_ratio retrieved the reference code in 161 cases (84.7%), exceeding token_sort_ratio with 148 cases (77.9%), partial_ratio with 147 cases (77.4%), and WRatio with 111 cases (58.4%).

At Top-30, token_set_ratio recovered the reference morphology code in 170 of the 190 evaluable records (89.5%; 95% CI, 84.3–93.1%). In comparison, partial_ratio, token_sort_ratio, and WRatio achieved retrieval rates of 80.5% (153/190), 78.4% (149/190), and 64.7% (123/190), respectively.

Expanding the candidate set generated by token_set_ratio from Top-20 to Top-30 recovered nine additional reference codes, corresponding to an absolute improvement of 4.7 percentage points. Based on its superior performance at the candidate-list depths selected for the hybrid experiments, token_set_ratio was retained as the lexical candidate-retrieval component of the proposed architecture. Complete Top-*N* retrieval results are presented in Table 1.

### 3.2. Ablation of Candidate-List Depth in the Hybrid Architecture

The influence of candidate-list depth on the performance of the proposed hybrid lexical–semantic architecture was evaluated by combining token_set_ratio candidate retrieval with LLM-based semantic code selection using Top-10, Top-20, and Top-30 candidate sets. Agreement, code-assignment, and confidence analyses were calculated using the 190 records containing sufficient morphological information for expert-reviewed reference coding.

The Top-10 configuration generated non-null morphology codes for 172 of the 190 evaluable diagnoses (90.5%) and returned null codes for 18 records (9.5%). The Top-20 configuration generated 179 non-null codes (94.2%) and 11 null responses (5.8%), whereas the Top-30 configuration generated 177 non-null codes (93.2%) and 13 null responses (6.8%).

**Figure 2:**
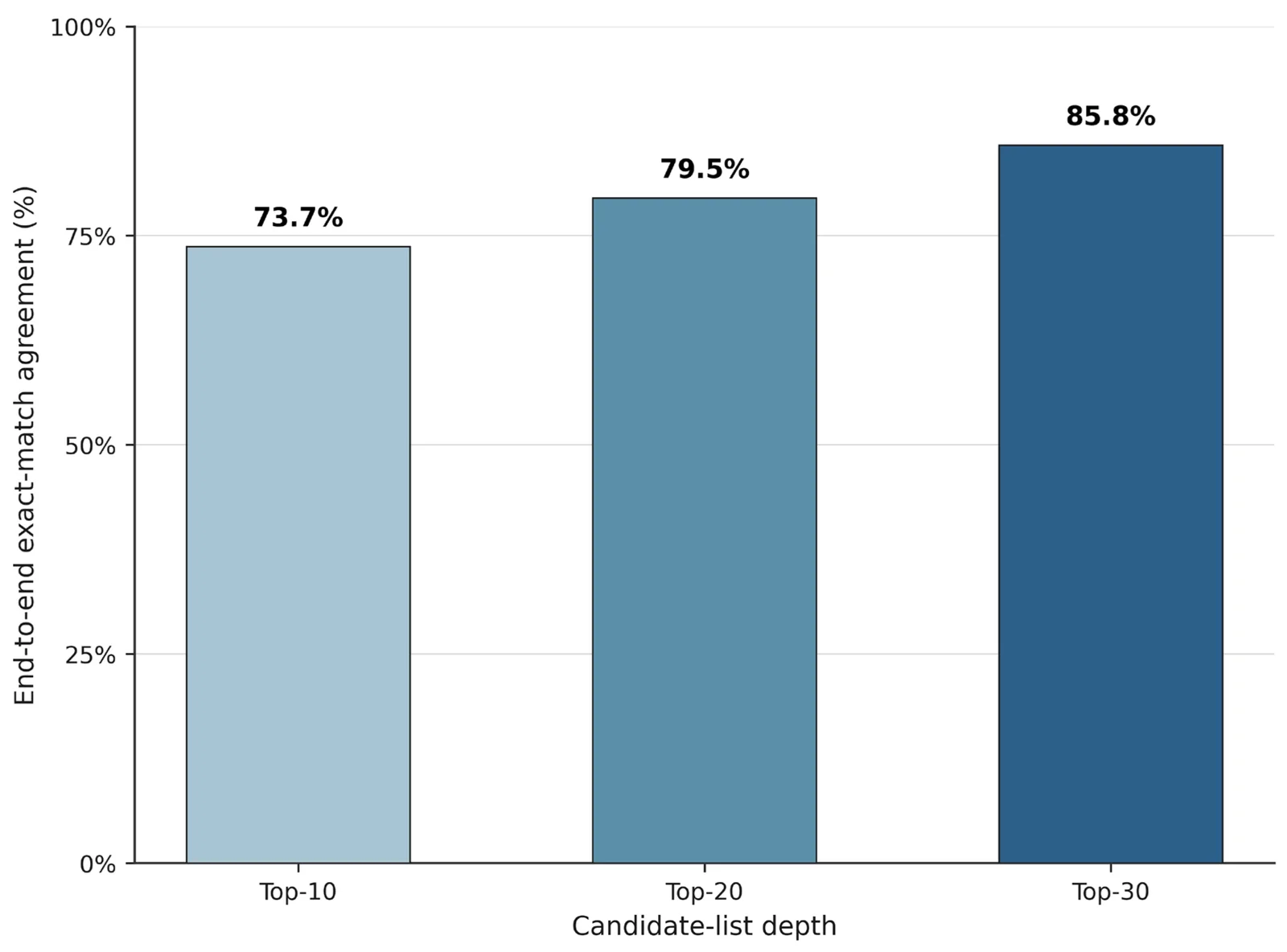
End-to-end exact-match agreement across hybrid candidate-list depths.

End-to-end exact-match agreement improved progressively as candidate-list depth increased. The Top-10 configuration correctly assigned the reference morphology code in 140 of the 190 evaluable records, corresponding to an agreement of 73.7% (95% CI, 67.0–79.4%). Agreement increased to 79.5% (151/190; 95% CI, 73.2–84.6%) for Top-20 and reached 85.8% (163/190; 95% CI, 80.1–90.0%) for Top-30. Overall, expanding the candidate set from Top-10 to Top-30 produced 23 additional exact matches, corresponding to an absolute improvement of 12.1 percentage points. The effect of candidate-list depth on end-to-end exact-match agreement is presented in Figure 2.

Conditional exact-match agreement reached 81.4% (140/172), 84.4% (151/179), and 92.1% (163/177; **95% CI, 87.2–95.2%**) for the Top-10, Top-20, and Top-30 configurations, respectively. Although Top-30 produced two fewer non-null outputs than Top-20, it generated 12 additional exact matches and improved end-to-end agreement by 6.3 percentage points.

To provide a more comprehensive evaluation beyond exact-match agreement, multiclass precision, recall, F1-score, and Cohen’s kappa were calculated for the Top-10, Top-20, and Top-30 configurations. Both macro-averaged and support-weighted metrics were reported to account for the unequal representation of morphology codes in the benchmark dataset. All metrics progressively improved with increasing candidate-list depth. Macro F1-score increased from 70.4% with Top-10 to 78.7% with Top-20 and 84.3% with Top-30, while weighted F1-score increased from 75.3% to 80.8% and 86.8%, respectively. Cohen’s κ similarly increased from 0.730 to 0.790 and 0.854. The complete multiclass performance results are presented in Table 2.

Inspection of the Top-30 results showed that the 27 non-concordant outcomes comprised 13 null predictions (abstentions) and 14 incorrect non-null morphology assignments. Among the 14 incorrect assignments, recurrent errors involved mast cell tumours (n = 3), mammary neoplasms (n = 3), and lymphomas (n = 3). Four misclassifications retained the same four-digit morphology base as the reference code but differed in the subtype extension, including one Sertoli-cell tumour code within the 8640 family and three mast cell tumour codes within the 9740 family. This pattern is consistent with the correct identification of the broader morphology family but incorrect resolution of the more specific subtype.

Component-level analysis of the final Top-30 configuration showed that the expert-reviewed reference code was present in the retrieved candidate set for 170 of 190 evaluable records (89.5%). Among these cases, 162 resulted in an exact final match, corresponding to 95.3% agreement when the reference code was successfully retrieved. Of the 27 non-concordant Top-30 outcomes, 19 (70.4%) occurred when the reference code was absent from the retrieved candidate set, whereas eight (29.6%) occurred despite successful retrieval. These eight cases comprised seven incorrect non-null predictions and one null output.

Other errors involved the selection of a related but less specific morphology code, including assignments in which the predicted code preserved the broader carcinoma category but did not resolve the expert-reviewed histological subtype. This pattern suggests that abbreviated or otherwise lexically underspecified diagnostic formulations may remain challenging.

To explore whether performance varied across morphology categories, the Top-30 end-to-end exact-match agreement was examined descriptively across morphology families defined according to the four-digit base of the Vet-ICD-O-Canine-1 morphology code. The five most frequently represented morphology families in the benchmark dataset are summarized in Table 3.

Performance varied across the five most frequently represented morphology families. Exact-match agreement was 76.2% for the 9740 family and 91.7% for the 8983 family, while the 8211, 8410, and 8941 families achieved 100% agreement within the present benchmark. Given the limited number of cases within individual morphology families, these results should be interpreted as descriptive subgroup findings rather than stable estimates of category-specific performance.

**Figure 3:**
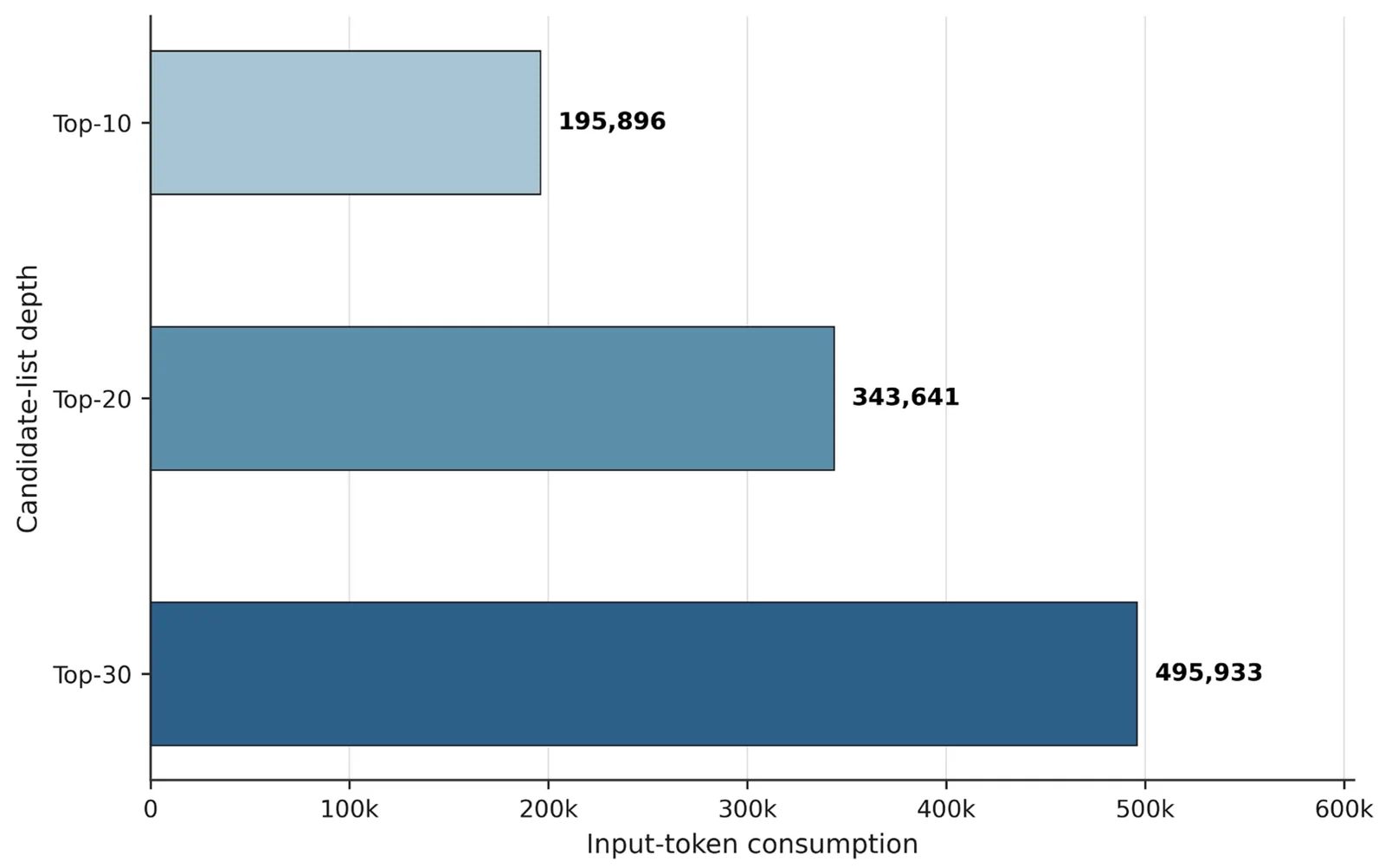
Trade-off between candidate-list depth and computational efficiency.

Increasing candidate-list depth was associated with higher computational requirements. Input-token consumption increased from 195,896 tokens with Top-10 to 343,641 with Top-20 and 495,933 with Top-30. Nevertheless, this increase was accompanied by consistent improvements in coding agreement, with Top-30 achieving the highest end-to-end and conditional exact-match agreement among the hybrid configurations. The relationship between candidate-list depth and input-token consumption is illustrated in Figure 3.

Model-reported confidence also increased with candidate-list depth. High-confidence predictions represented 72.1% (137/190) of the evaluable records in the Top-10 configuration, increasing to 75.3% (143/190) with Top-20 and 80.0% (152/190) with Top-30. These confidence labels were treated as descriptive model outputs and were not interpreted as calibrated probabilities of prediction correctness.

Model-reported confidence was also associated with observed coding agreement. In the Top-30 configuration, 145 of 152 high-confidence outputs were exact matches (95.4%), compared to 18 of 25 medium-confidence outputs (72.0%). All 13 low-confidence outputs corresponded to null predictions and therefore did not yield an exact morphology-code match. Similar patterns were observed for Top-10 and Top-20, with high-confidence exact-match rates of 92.7% and 93.7%, respectively, compared to 37.1% and 47.2% among medium-confidence outputs. These findings indicate that the categorical confidence labels were associated with observed agreement in this benchmark; however, they should not be interpreted as calibrated probabilities of correctness.

The 13 null outputs observed among the 190 evaluable diagnoses in the Top-30 configuration included abbreviations, compound diagnostic statements, and descriptions containing multiple morphological or contextual modifiers. Null outputs were recurrent among selected morphology groups, including transitional cell carcinomas and squamous cell carcinomas. Component-level error attribution showed that 12 of the 13 null outputs occurred when the expert-reviewed reference code was absent from the Top-30 candidate set, whereas one null output occurred despite successful retrieval. Thus, most null outputs were associated with candidate-retrieval failure rather than semantic abstention.

### 3.3. Computational Efficiency of the Hybrid Architecture

Computational requirements increased with candidate-list depth but remained substantially lower than those of direct LLM inference using the complete Vet-ICD-O-Canine-1 morphology ontology. Resource consumption was recorded across the complete set of 211 submitted diagnoses, including both the 190 evaluable diagnoses and the 21 insufficiently specified records.

The Top-10 hybrid configuration consumed 195,896 input tokens and 14,079 output tokens, totalling 209,975 tokens, at an inference cost of USD 0.27. The Top-20 configuration consumed 343,641 input tokens and 14,356 output tokens, totalling 357,997 tokens, at a cost of USD 0.41. The Top-30 configuration consumed 495,933 input tokens and 14,368 output tokens, totalling 510,301 tokens, at an inference cost of USD 0.57.

Compared to Top-20, expanding the candidate set to Top-30 required an additional 152,292 input tokens and increased inference cost by USD 0.16. This additional computational investment was accompanied by 12 additional exact matches among the 190 evaluable diagnoses.

**Figure 4:**
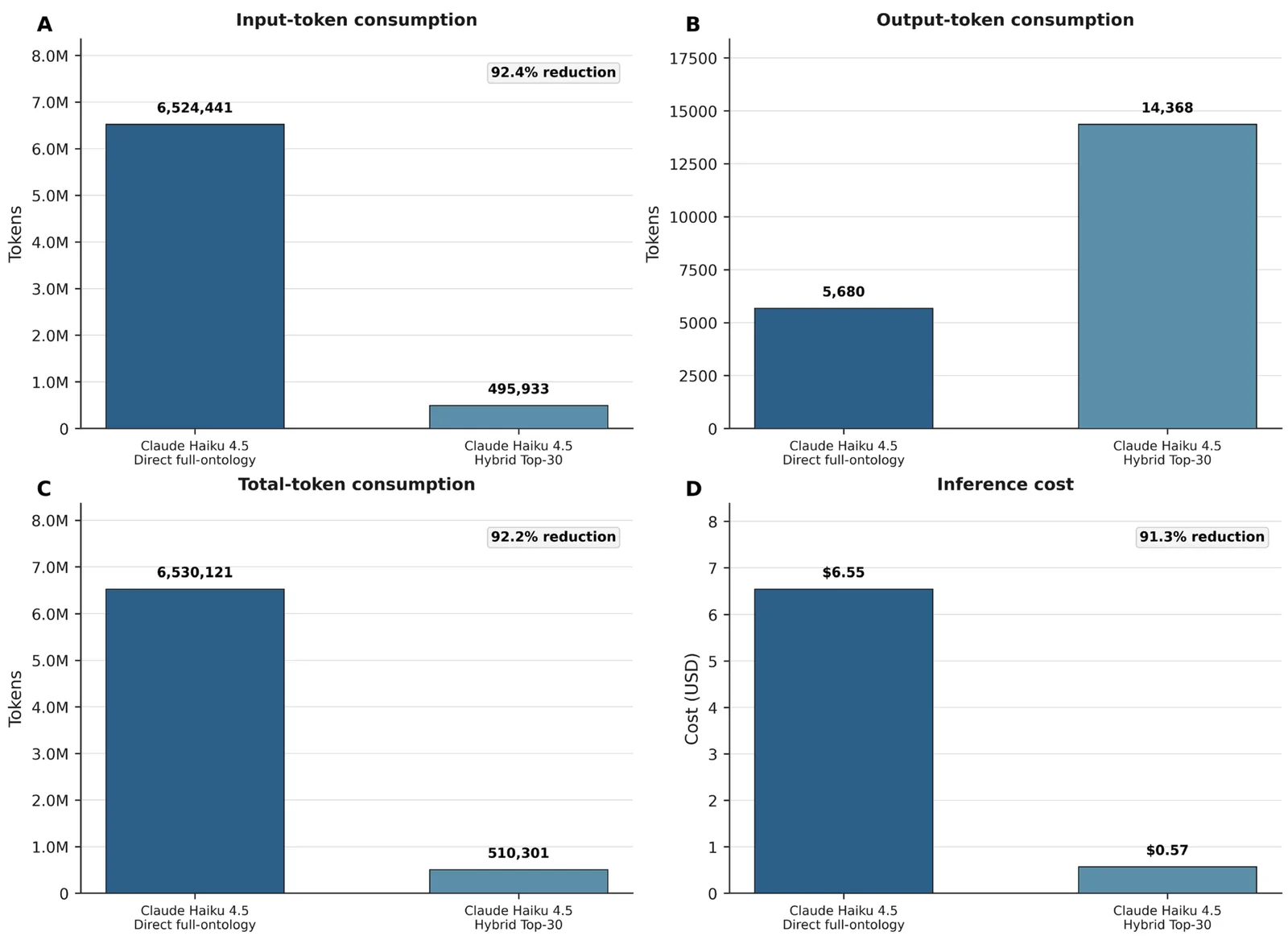
Computational efficiency of direct and retrieval-assisted LLM inference for Vet-ICD-O-Canine-1 morphology coding.

In contrast, direct LLM inference using the complete Vet-ICD-O-Canine-1 morphology ontology required 6,524,441 input tokens and 5680 output tokens, totalling 6,530,121 tokens, at an inference cost of USD 6.55. Relative to the direct full-ontology baseline, Top-30 reduced input-token consumption by 92.4%, total token consumption by 92.2%, and inference cost by 91.3%. The comparison between direct full-ontology inference and the Top-30 hybrid configuration is presented in Figure 4.

### 3.4. Comparison with Direct Full-Ontology LLM Inference

In the direct baseline configuration, each of the 211 diagnoses was submitted together with the complete Vet-ICD-O-Canine-1 morphology ontology comprising 971 codes. Across all submitted records, 118 requests encountered API rate-limit errors and were recorded as technical execution failures.

Among the 190 diagnoses with expert-reviewed reference codes, 106 requests encountered technical failures, 25 returned null codes, and 59 generated non-null morphology-code predictions. Of the 59 non-null predictions, 42 were concordant and 17 were discordant with the expert-reviewed reference standard.

Conditional exact-match agreement among the non-null predictions was therefore 71.2% (42/59; 95% CI, 58.6–81.2%). Considering all 190 evaluable diagnoses, with incorrect predictions, null outputs, and technical failures treated as non-concordant outcomes, the direct baseline achieved an end-to-end exact-match agreement of 22.1% (42/190; 95% CI, 16.8–28.5%).

In comparison, the Top-30 hybrid configuration generated non-null morphology codes for 177 of the 190 evaluable diagnoses (93.2%) and correctly assigned the reference code in 163 cases. This corresponded to a conditional exact-match agreement of 92.1% (163/177) and an end-to-end exact-match agreement of 85.8% (163/190).

Top-30 therefore increased end-to-end exact-match agreement by 63.7 percentage points relative to the direct baseline. No API rate-limit errors occurred in any of the retrieval-assisted Top-10, Top-20, or Top-30 configurations.

Overall, retrieval-assisted inference substantially improved operational robustness, non-null code-assignment availability, and agreement with the expert-reviewed reference standard while reducing inference context, token consumption, and cost. However, the Top-30 configuration still returned null codes for 13 evaluable diagnoses, indicating that candidate coverage and semantic abstention remain relevant areas for further investigation.

### 3.5. Secondary Exploratory Analysis of Insufficiently Specified Diagnoses

The 21 records containing generic or non-specific descriptions, such as “tumour” or “neoplasia”, were processed under each experimental configuration. These records were excluded from candidate-retrieval and exact-match agreement analyses because they did not contain sufficient morphological information for the assignment of an unambiguous expert-reviewed Vet-ICD-O-Canine-1 reference code.

The records were retained as a secondary exploratory challenge subset to evaluate model behaviour when the available diagnostic information was insufficient for unambiguous morphology coding. A null morphology code was interpreted as an appropriate abstention. A non-null code was recorded as a code assignment on an insufficiently specified input; because no unambiguous reference code existed, the correctness of these assignments could not be formally evaluated. Technical execution failures were recorded separately.

The Top-10 configuration returned null codes in 10 of the 21 records (47.6%) and generated non-null morphology codes in 11 cases (52.4%). Top-20 returned null codes in nine records (42.9%) and generated codes in 12 cases (57.1%). Top-30 returned null codes in eight records (38.1%) and generated codes in 13 cases (61.9%). No technical execution failures occurred in any retrieval-assisted configuration. The comparative results for each configuration are summarized in Table 4.

In the direct full-ontology configuration, 12 of the 21 requests encountered technical failures (57.1%). Six records returned null codes (28.6% of all 21 records), and three generated non-null morphology codes (14.3%). When only the nine technically completed requests were considered, six resulted in null codes (66.7%) and three generated non-null codes (33.3%).

These exploratory findings revealed a trade-off between coding performance among sufficiently specified diagnoses and abstention behaviour among insufficiently specified inputs. Increasing candidate-list depth improved exact-match agreement in the primary analysis but was accompanied by a progressive reduction in null responses within the challenge subset.

### 3.6. Selection of the Final Hybrid Architecture

Among the evaluated retrieval-assisted configurations, Top-30 achieved the highest end-to-end exact-match agreement (85.8%), the highest conditional exact-match agreement (92.1%), and the highest proportion of high-confidence predictions (80.0%) among diagnoses containing sufficient morphological information for expert-reviewed reference coding.

The Top-30 architecture generated non-null codes for 177 of the 190 evaluable diagnoses (93.2%), produced no API rate-limit failures across the complete set of 211 submitted records, and maintained substantially lower token consumption and inference cost than direct full-ontology inference.

Accordingly, Top-30 was selected as the final hybrid lexical–semantic configuration based on its performance in the primary reference-standard analysis and its computational efficiency. The final architecture consisted of a two-stage workflow in which token_set_ratio first retrieved the 30 most lexically similar Vet-ICD-O-Canine-1 morphology candidates, followed by LLM-based semantic reasoning to select the final morphology code or return a null response.

The secondary exploratory analysis of insufficiently specified descriptions nevertheless showed that the final configuration generated non-null codes for 61.9% of these records. This finding indicates that, although Top-30 provided the most favourable balance for morphology-code selection among codifiable diagnoses, an additional data-sufficiency assessment or abstention-control mechanism would be required before autonomous operational deployment.

## 4. Discussion

### 4.1. Principal Findings

This study developed and evaluated a hybrid lexical–semantic architecture for the automated assignment of Vet-ICD-O-Canine-1 morphology codes from free-text veterinary pathology diagnoses. The proposed approach separated lexical candidate retrieval from LLM-based semantic selection, reducing the number of ontology entries presented to the model while preserving the original diagnostic text for contextual interpretation.

The primary analysis included 190 diagnoses containing sufficient morphological information for assignment of an expert-reviewed reference code. Among the evaluated hybrid configurations, Top-30 achieved the highest end-to-end exact-match agreement, correctly assigning the reference morphology code in 163 of 190 cases (85.8%). It generated non-null codes for 177 cases (93.2%), resulting in a conditional exact-match agreement of 92.1% among completed code assignments. The Top-30 configuration also produced the highest proportion of model-reported high-confidence predictions, although these confidence labels were not formally calibrated.

These improvements were accompanied by substantial gains in computational efficiency. Relative to direct LLM inference over the complete 971-entry morphology ontology, Top-30 reduced input-token consumption by 92.4%, total token consumption by 92.2%, and inference cost by 91.3%. No API rate-limit failures occurred in the retrieval-assisted configurations, whereas the full-ontology baseline encountered 118 technical execution failures across the 211 submitted records. These findings indicate that candidate reduction improved both coding performance and operational robustness under the experimental conditions evaluated.

A secondary exploratory analysis assessed the behaviour of the architectures on 21 generic or insufficiently specified descriptions, such as “tumour” or “neoplasia”, for which no unambiguous reference morphology code could be assigned. In this subset, the Top-30 configuration returned null codes in eight of 21 cases (38.1%) and generated non-null codes in 13 cases (61.9%). This finding does not alter the primary agreement results, because these records were not included in the 190-case reference-standard analysis. However, it identifies an important distinction between two separate tasks: selecting the correct morphology code when sufficient diagnostic information is available and determining whether the input contains enough information to be coded at all.

The final architecture was therefore most effective as a morphology-code selection system for sufficiently specified diagnoses. Its current components were not sufficient to ensure reliable abstention when the diagnostic description lacked the information required for unambiguous morphology coding.

### 4.2. Comparison with Previous Studies

Automated processing of pathology narratives has evolved from manually engineered rules and conventional NLP methods toward transformer-based language models and compound AI architectures. Previous oncology applications have included information extraction, identification of reportable tumours, cancer registry abstraction, and standardized coding from unstructured clinical text [1,2].

Hammami et al. developed a rule-based NLP system for assigning ICD-O morphology codes from Italian pathology reports and demonstrated that highly accurate automated coding can be achieved through manually designed linguistic rules [19]. Rule-based methods offer transparency and can perform well within the reporting environment for which they were developed. Nevertheless, they generally require language-specific dictionaries, explicit linguistic patterns, and continued adaptation when terminology or institutional reporting practices change. The present architecture addressed this limitation by using fuzzy lexical retrieval to accommodate variation in wording and an LLM to perform contextual discrimination among candidate concepts.

Direct numerical comparison with the rule-based study would not be appropriate because of differences in language, dataset size, report structure, coding targets, ontology coverage, and evaluation design. Nevertheless, both studies support the feasibility of transforming heterogeneous pathology narratives into standardized oncology codes. The main difference is that the present approach distributes the task across retrieval and semantic-selection components rather than encoding the complete decision process in manually constructed rules.

More recent studies have investigated LLM-based cancer registry coding. Wang et al. evaluated a retrieval-augmented system for cancer registration using longitudinal hospital records [27]. In that architecture, retrieval was used to identify relevant patient-specific information distributed across clinical documents. In the present study, retrieval served a different purpose: it selected plausible ontology concepts from the Vet-ICD-O-Canine-1 morphology vocabulary before semantic classification. The current method should therefore be understood as retrieval-assisted ontology coding rather than conventional retrieval-augmented generation of clinical answers.

Arzideh et al. evaluated self-hosted language models for ICD-O coding from real-world human pathology reports [28]. Their work demonstrated the feasibility of using LLMs for oncology classification while addressing concerns associated with external model hosting. The present study examined a complementary architectural question of whether inexpensive lexical candidate reduction can improve coding performance, computational efficiency, and operational reliability regardless of the broader hosting strategy.

Reviews of LLM applications in oncology emphasize their potential for information extraction and clinical data standardization, while also identifying concerns related to generalizability, hallucinations, privacy, transparency, and inappropriate confidence in outputs [25]. The results of the 21-record challenge subset reinforce this concern. Even when a diagnostic description did not support unambiguous morphology coding, the model frequently selected one of the available candidates. This behaviour illustrates why successful code selection and safe abstention should be evaluated separately.

In veterinary medicine, language models have already been investigated for diagnostic prediction from clinical notes [26], and veterinary pathology has been identified as an area with considerable potential for NLP-based data extraction and standardization [15,16]. The present study extends this emerging literature by focusing specifically on ontology-based morphology coding for veterinary cancer registries and by evaluating predictive performance together with computational consumption, technical failures, null outputs, and behaviour on insufficiently specified inputs.

### 4.3. Mechanistic Interpretation of the Hybrid Architecture

The performance of the proposed architecture can be interpreted as the result of a staged reduction in decision complexity. Direct full-ontology inference required the LLM to compare each diagnosis against 971 morphology entries, many of which were lexically or semantically related. In the hybrid configurations, the lexical stage reduced this broad search problem to a smaller set of plausible candidates, allowing the semantic component to focus on contextual distinctions among closely related concepts.

The fuzzy-matching results demonstrate that retrieval methods should be selected according to their intended architectural role. When fuzzy matching was used as a standalone Top-1 classifier, token_sort_ratio achieved the highest exact-match performance. However, token_set_ratio achieved superior reference-code coverage from Top-10 onward and recovered the correct code within the Top-30 candidate set in 170 of the 190 evaluable cases. Thus, the most effective standalone lexical classifier was not the most effective upstream retriever.

This distinction is important because the retrieval component is not expected to make the final semantic decision. Its primary objective is to retain the correct ontology concept within a sufficiently compact candidate set. The LLM subsequently evaluates the original diagnosis in relation to those candidates and selects the final morphology code. The architecture therefore assigns complementary responsibilities to the two components: lexical similarity reduces the search space, whereas semantic reasoning resolves contextual ambiguity.

The approach is conceptually related to retrieval-augmented generation, in which relevant external information is selected before model inference [31]. However, the retrieved elements in this study were not external clinical documents or factual passages. They were allowable ontology concepts. Retrieval therefore constrained the semantic decision space rather than supplying additional patient information.

This distinction may explain the substantial reduction in computational demand. Previous research has shown that simply increasing context length does not guarantee that all included information will be used effectively, especially when the relevant item is surrounded by numerous competing entries [30]. Retrieval-based and long-context approaches consequently involve different trade-offs between information coverage, computational requirements, and model attention [32]. Hybrid retrieval strategies similarly seek to combine efficient candidate identification with more computationally intensive reasoning only where it is most useful [33].

Candidate-list depth represented a central architectural trade-off. Increasing the candidate set from Top-10 to Top-30 improved end-to-end agreement from 73.7% to 85.8%, indicating that narrower candidate sets frequently omitted concepts needed for correct downstream classification. Nevertheless, increasing the number of candidates also increased token consumption and exposed the model to a larger number of plausible alternatives.

The exploratory challenge analysis revealed an additional consequence of increasing candidate-list depth. Null responses decreased from 47.6% with Top-10 to 38.1% with Top-30 among the 21 insufficiently specified inputs. One possible interpretation is that larger candidate sets increased the probability that the model would identify a superficially plausible morphology concept even when the diagnostic text did not contain enough information for an unambiguous classification. This is an inference from the observed pattern and should be evaluated through dedicated experiments rather than regarded as a confirmed causal mechanism.

Component-level analysis indicated that candidate coverage was an important determinant of end-to-end performance. Among the 27 non-concordant Top-30 outcomes, 19 (70.4%) occurred when the expert-reviewed reference code was absent from the retrieved candidate set, whereas eight (29.6%) occurred despite successful retrieval. When the reference code was retrieved, 162 of 170 cases (95.3%) resulted in an exact final match. These findings suggest that improving candidate retrieval may address a substantial proportion of the remaining errors, although downstream semantic selection and abstention also contributed to non-concordant outcomes.

The comparison with the full-ontology baseline should also be interpreted cautiously. Its end-to-end agreement of 22.1% incorporated incorrect predictions, null responses, and technical execution failures. Therefore, it represents overall operational performance under the evaluated API conditions rather than a pure estimate of the intrinsic semantic capacity of unrestricted long-context inference. Nevertheless, the higher conditional agreement of Top-30 and the absence of rate-limit errors support the practical value of candidate reduction within the tested environment.

### 4.4. Practical Implications for Veterinary Cancer Registries

Veterinary cancer registries depend on the transformation of heterogeneous diagnostic narratives into standardized, interoperable, and reusable data [11,14]. Automated morphology coding could reduce the manual effort required for retrospective data harmonization and prospective case registration, supporting epidemiological surveillance, institutional comparisons, and comparative oncology research.

The proposed architecture could be integrated into a registry workflow after the relevant diagnostic text has been extracted from the pathology report. The lexical stage can identify plausible morphology concepts, while the semantic component selects the most appropriate code and returns it in a structured format. This output could subsequently be evaluated through database validation rules or presented to a cancer registrar for confirmation.

The structured output format also facilitates auditability. An operational implementation could retain the original diagnostic description, the retrieved candidates, the selected morphology code, the model-reported confidence label, the model and ontology versions, and any human correction. Such records would allow errors to be investigated and would support periodic reassessment of system performance.

However, the challenge-subset results indicate that this workflow requires an additional step before candidate retrieval. A practical system should first determine whether the diagnostic description contains sufficient morphological information for coding. Records containing only terms such as “tumour”, “neoplasia”, or similarly generic descriptions should be routed for manual review or returned as insufficiently specified rather than submitted directly for code selection.

A safer operational architecture would therefore contain at least three distinct decision stages:

1. Assessment of whether the input contains sufficient information for morphology coding;
2. Retrieval and semantic selection of a morphology code when the input is considered codifiable;
3. Human review of abstentions, ambiguous cases, low-confidence outputs, or cases that fail predefined validation rules.

The present study primarily evaluated the second stage. The exploratory analysis of the 21 insufficiently specified diagnoses demonstrates why the first stage cannot be assumed to emerge automatically from candidate retrieval and semantic selection.

This distinction is also relevant to companion-animal cancer data parsing initiatives [36]. Parsing and coding systems must not only extract information but also preserve uncertainty and distinguish absent information from negative findings or generic diagnostic terminology. For registries such as the São Paulo Animal Cancer Registry [35], an explicit sufficiency-assessment component could reduce the risk of introducing apparently precise morphology codes derived from inadequately specified source data.

Accordingly, the proposed architecture should be considered a decision-support and data-standardization tool rather than an autonomous replacement for veterinary pathologists or cancer registrars. Human oversight remains especially important for uncommon morphologies, ambiguous terminology, conflicting information, and descriptions that do not support a unique code assignment. In an operational registry workflow, veterinary pathologists or trained cancer registry personnel should review flagged cases and retain responsibility for validating the final morphology code before its incorporation into the registry.

### 4.5. Strengths and Limitations

A major strength of this study was the use of original diagnostic descriptions without manual normalization, simplification, removal of modifiers, or adaptation to ontology terminology. This preserved the lexical and contextual variability encountered in routine veterinary pathology data. The evaluable dataset included 170 unique descriptions spanning 74 distinct reference morphology codes, allowing the architecture to be assessed across diverse diagnostic formulations within the morphology categories represented in the benchmark.

A second strength was the component-based experimental design. Fuzzy metrics were evaluated both as standalone classifiers and as candidate-retrieval methods, demonstrating that these roles require different criteria for algorithm selection. The comparison among Top-10, Top-20, and Top-30 configurations constituted an explicit ablation of candidate-list depth. Direct full-ontology inference provided an operational baseline against which the effects of retrieval on agreement, token consumption, cost, and technical execution could be evaluated.

The processing of all 211 records represents an additional strength. Rather than discarding generic or insufficiently specified descriptions entirely, the study retained 21 such records for a secondary exploratory assessment of model behaviour. This analysis exposed an important limitation that would not have been identified through exact-match evaluation alone: high performance among codifiable records does not necessarily imply reliable abstention among non-codifiable inputs.

Several limitations should nevertheless be considered. First, the primary reference-standard analysis included 190 diagnoses from a single cancer registry. External validation using data from other laboratories, geographic regions, languages, species, pathologists, and reporting systems is required. Although the architecture searched the complete 971-entry Vet-ICD-O-Canine-1 morphology ontology, only 74 morphology codes were represented by expert-reviewed reference cases in the benchmark dataset. Consequently, performance could not be empirically established for morphology categories absent from the benchmark, and estimates for infrequently represented codes may be less precise.

Second, the 21-record challenge subset was small and exploratory. It was not designed to provide a formal estimate of specificity, false-positive coding, or safety. Because no unambiguous reference morphology codes existed for these records, the non-null assignments cannot be evaluated as correct or incorrect through exact matching. They can only be interpreted as code assignments made despite insufficient information for a definitive reference classification.

Third, the criteria used to identify the 21 insufficiently specified diagnoses were based on the inability to assign an unambiguous expert-reviewed morphology code. Future work should formalize these criteria and assess inter-reviewer agreement. A more diverse challenge set should include generic terminology, incomplete diagnoses, conflicting statements, abbreviations, non-neoplastic findings, and descriptions referring only to anatomical location or tumour behaviour.

Fourth, the study evaluated morphology coding only. It did not address topography, laterality, behaviour, grade, stage, or the extraction of information distributed across different sections of a complete pathology report. The model received the final diagnostic description rather than the complete report, which limited the task to concept normalization based on the information contained in that field.

Fifth, semantic selection was evaluated using a single commercial model and a fixed model version. Performance may differ with other proprietary, open-source, smaller, or locally deployed models. API prices, rate limits, and model availability may also change, meaning that the reported costs and technical failures should be interpreted as measurements under the specific experimental conditions. Future studies should compare the finalized retrieval architecture across proprietary and open-source LLMs under identical candidate sets, prompts, and inference conditions to determine the extent to which the observed performance is model-dependent.

Sixth, model-reported confidence labels were not calibrated and should not be interpreted as probabilities of correctness. Their use for automatic case acceptance would require formal calibration against observed agreement and predefined safety thresholds.

Finally, the component-level error analysis was descriptive and was based on only 27 non-concordant Top-30 outcomes. Although this analysis distinguished failures associated with the absence of the reference code from those occurring despite successful retrieval, the limited number of errors precluded stable estimates for specific morphology categories or textual characteristics. Larger external datasets will be required to characterize recurrent retrieval and semantic-selection failure patterns more precisely.

### 4.6. Future Directions

The first priority for future research is external validation using multicenter and multilingual veterinary pathology datasets. Although the present study used real-world diagnoses collected by a veterinary cancer registry, the architecture was evaluated retrospectively rather than within an active prospective registry workflow. Future evaluation should therefore examine performance across laboratories, pathologists, geographic regions, species, tumour distributions, and institutional terminology. A prospective registry study would additionally allow the measurement of processing time, reviewer workload, correction frequency, and the effect of the system on final registry data quality.

A dedicated data-sufficiency component should be developed and evaluated before operational deployment. Potential approaches include explicit rules for generic diagnostic terms, a separate classifier trained to distinguish codifiable from non-codifiable descriptions, calibrated abstention thresholds, or a preliminary LLM instruction focused specifically on information sufficiency. This component should operate before ontology candidate retrieval so that insufficient descriptions are not forced into a code-selection task.

Future studies should extend component-level error analysis in larger external datasets by examining the lexical rank of the reference code, the semantic alternatives selected by the model, and the characteristics of cases associated with retrieval failure, semantic misselection, or abstention. Such analyses could guide targeted improvements in both candidate retrieval and downstream semantic selection.

Alternative retrieval strategies should be compared with token_set_ratio, including conventional information-retrieval methods, multilingual embeddings, dense semantic retrieval, cross-encoder reranking, ontology-hierarchy information, and combinations of lexical and semantic signals. Candidate-list depth should also be optimized on independent development data rather than assumed to be universally fixed at Top-30.

The architecture should be evaluated with other language models, including smaller and locally deployed models. Candidate reduction may allow less computationally intensive models to achieve acceptable performance while improving privacy, reproducibility, and control over inference infrastructure.

Extension to other Vet-ICD-O-Canine-1 dimensions is also warranted. Topography, behaviour, laterality, grade, and other cancer registry variables may require separate extraction and coding components. A modular architecture could process each dimension independently while sharing sufficiency assessment, structured output validation, audit records, and human-review mechanisms.

Finally, prospective deployment should include model and ontology versioning, monitoring of performance drift, calibrated human-review thresholds, documentation of corrections, and periodic reassessment. These system-level safeguards are necessary to translate retrospective coding performance into a reliable cancer registry workflow [25,36,37].

## 5. Conclusions

This study developed and evaluated a hybrid lexical–semantic architecture for the automated assignment of Vet-ICD-O-Canine-1 morphology codes from free-text veterinary pathology diagnoses. By combining fuzzy lexical candidate retrieval with LLM-based semantic selection, the proposed approach reduced the ontology search space presented to the model while preserving contextual interpretation of the original diagnostic text.

Among the 190 diagnoses containing sufficient morphological information for expert-reviewed reference coding, the Top-30 configuration achieved an end-to-end exact-match agreement of 85.8% and a conditional agreement of 92.1% among non-null predictions. For comparison, the direct full-ontology baseline achieved 71.2% conditional exact-match agreement among non-null predictions but only 22.1% end-to-end exact-match agreement when incorrect predictions, null outputs, and technical execution failures were considered non-concordant outcomes. It generated non-null morphology codes for 93.2% of evaluable diagnoses and, relative to direct inference over the complete 971-record computational representation of the morphology ontology, reduced input-token consumption by 92.4%, total token consumption by 92.2%, and inference cost by 91.3%. The retrieval-assisted configurations also avoided the API rate-limit failures observed with direct full-ontology inference under the evaluated experimental conditions.

The secondary exploratory analysis of 21 insufficiently specified diagnoses showed that the final architecture did not reliably abstain when the input lacked enough information for unambiguous morphology coding. Therefore, the proposed system is best interpreted as a code-selection and data-standardization approach for sufficiently specified diagnostic descriptions rather than as a fully autonomous coding system. External validation in larger independent datasets, calibrated abstention mechanisms, and an upstream assessment of diagnostic sufficiency are required before operational implementation, while further component-level error analyses should be conducted to characterize failure patterns across more diverse settings. Nevertheless, the findings support ontology-guided candidate reduction as a promising strategy for improving the accuracy, computational efficiency, and operational robustness of automated veterinary cancer registry coding. These findings may be particularly relevant to veterinary cancer registries, cancer registrars, veterinary pathologists, and researchers developing automated data-standardization pipelines for veterinary and comparative oncology.

## Figures and Tables

**Table 1:** Candidate-retrieval performance of fuzzy similarity metrics.

Fuzzy Metric	Top-1	Top-3	Top-5	Top-10	Top-20	Top-30	Top-50	Top-100
token_set_ratio	44.2% (84/190)	57.9% (110/190)	65.3% (124/190)	75.8% (144/190)	84.7% (161/190)	89.5% (170/190)	93.7% (178/190)	94.7% (180/190)
token_sort_ratio	47.9% (91/190)	60.5% (115/190)	66.3% (126/190)	73.2% (139/190)	77.9% (148/190)	78.4% (149/190)	82.6% (157/190)	86.3% (164/190)
WRatio	33.7% (64/190)	45.3% (86/190)	47.4% (90/190)	52.1% (99/190)	58.4% (111/190)	64.7% (123/190)	70.0% (133/190)	85.8% (163/190)
partial_ratio	38.4% (73/190)	50.0% (95/190)	53.2% (101/190)	62.1% (118/190)	77.4% (147/190)	80.5% (153/190)	83.7% (159/190)	90.5% (172/190)

**Table 2:** Multiclass performance metrics across hybrid candidate-list configurations.

Candidate-List Depth	Exact Match	Macro Precision	Macro Recall	Macro F1	Weighted Precision	Weighted Recall	Weighted F1	Cohen’s κ
Top-10	73.7%	73.9%	72.1%	70.4%	82.5%	73.7%	75.3%	0.730
Top-20	79.5%	81.7%	79.5%	78.7%	87.4%	79.5%	80.8%	0.790
Top-30	85.8%	86.0%	85.2%	84.3%	91.7%	85.8%	86.8%	0.854

**Note:** Macro-averaged metrics assign equal weight to each of the 74 morphology codes represented in the reference-standard dataset, whereas weighted metrics are weighted according to class support. Null outputs were considered non-concordant outcomes but were not treated as morphology classes for precision, recall, and F1-score calculations. Cohen’s κ was calculated across all 190 evaluable records, retaining null outputs as a distinct predicted outcome.

**Table 3:** Exact-match agreement of the Top-30 hybrid configuration across the five most frequent morphology families.

Morphology Family	N	Exact Matches	Exact-Match Agreement
9740	21	16	76.2%
8211	14	14	100%
8410	14	14	100%
8983	12	11	91.7%
8941	11	11	100%

**Note:** Morphology families were defined according to the four-digit base of the Vet-ICD-O-Canine-1 morphology code. The five families with the highest number of reference-standard cases are shown. Exact-match agreement represents the proportion of cases within each family for which the Top-30 configuration returned the expert-reviewed reference morphology code. These subgroup results are descriptive and should be interpreted cautiously because of the limited number of observations within individual categories.

**Table 4:** Behaviour of the evaluated configurations on insufficiently specified diagnostic descriptions.

Output Category	Top-10	Top-20	Top-30	Direct Full-Ontology
Non-null code assignment	52.4% (11/21)	57.1% (12/21)	61.9% (13/21)	14.3% (3/21)
Null code	47.6% (10/21)	42.9% (9/21)	38.1% (8/21)	28.6% (6/21)
Technical failure	0% (0/21)	0% (0/21)	0% (0/21)	57.1% (12/21)
Total	21	21	21	21

## Data Availability

The individual veterinary pathology records used in this study are not publicly available because of institutional data-governance restrictions applicable to the source cancer registry. Access to the study data may be considered upon reasonable request to the corresponding author, subject to institutional approval and applicable data-governance requirements. The computational code and associated implementation files are not publicly available at this stage because they form part of an ongoing doctoral research project; however, they may also be made available upon reasonable request to the corresponding author for academic and reproducibility purposes. Public release of the computational materials is planned following the completion of the broader doctoral research project, subject to applicable institutional policies.

## Abbreviations

The following abbreviations are used in this manuscript:

AI	Artificial Intelligence
API	Application Programming Interface
CI	Confidence Interval
CSV	Comma-Separated Values
GPU	Graphics Processing Unit
HTTP	Hypertext Transfer Protocol
ICD-O	International Classification of Diseases for Oncology
JSON	JavaScript Object Notation
LLM	Large Language Model
NLP	Natural Language Processing
RAM	Random-Access Memory
USD	United States Dollar
Vet-ICD-O-Canine-1	Veterinary International Classification of Diseases for Oncology Canine Tumours, First Edition
